# A Novel Integrated Metabolism-Immunity Gene Expression Model Predicts the Prognosis of Lung Adenocarcinoma Patients

**DOI:** 10.3389/fphar.2021.728368

**Published:** 2021-07-30

**Authors:** Songming Chen, Yumei Duan, Yanhao Wu, Desong Yang, Jian An

**Affiliations:** ^1^ Key Laboratory of Molecular Radiation Oncology Hunan Province, Xiangya Hospital, Central South University, Changsha, China; ^2^ Xiangya Cancer Center, Xiangya Hospital, Central South University, Changsha, China; ^3^ Department of Pathology, Xiangya Hospital, Central South University, Changsha, China; ^4^ Department of Respiratory Medicine, Xiangya Hospital, Central South University, Changsha, China; ^5^ Thoracic Surgery Department II, Hunan Cancer Hospital & the Affiliated Cancer Hospital of Xiangya School of Medicine, Central South University, Changsha, China; ^6^ National Key Clinical Specialty, Branch of National Clinical Research Center for Respiratory Disease, Xiangya Hospital, Central South University, Changsha, China; ^7^ Xiangya Lung Cancer Center, Xiangya Hospital, Central South University, Changsha, China; ^8^ Hunan Provincial Clinical Research Center for Respiratory Diseases, Changsha, China

**Keywords:** lung adenocarcinoma, tumor microenvironment, TYMS, NME4, LDHA, SMOX, CD19, CD68

## Abstract

**Background:** Although multiple metabolic pathways are involved in the initiation, progression, and therapy of lung adenocarcinoma (LUAD), the tumor microenvironment (TME) for immune cell infiltration that is regulated by metabolic enzymes has not yet been characterized.

**Methods:** 517 LUAD samples and 59 non-tumor samples were obtained from The Cancer Genome Atlas (TCGA) database as the training cohort. Kaplan-Meier analysis and Univariate Cox analysis were applied to screen the candidate metabolic enzymes for their role in relation to survival rate in LUAD patients. A prognostic metabolic enzyme signature, termed the metabolic gene risk score (MGRS), was established based on multivariate Cox proportional hazards regression analysis and was verified in an independent test cohort, GSE31210. In addition, we analyzed the immune cell infiltration characteristics in patients grouped by their Risk Score. Furthermore, the prognostic value of these four enzymes was verified in another independent cohort by immunohistochemistry and an optimized model of the metabolic-immune protein risk score (MIPRS) was constructed.

**Results:** The MGRS model comprising 4 genes (*TYMS, NME4, LDHA*, and *SMOX*) was developed to classify patients into high-risk and low-risk groups. Patients with a high-risk score had a poor prognosis and exhibited activated carbon and nucleotide metabolism, both of which were associated with changes to TME immune cell infiltration characteristics. In addition, the optimized MIPRS model showed more accurate predictive power in prognosis of LUAD.

**Conclusion:** Our study revealed an integrated metabolic enzyme signature as a reliable prognostic tool to accurately predict the prognosis of LUAD.

## Introduction

Lung cancer, one of the most prominent malignant tumors, has the highest mortality rate in humans worldwide. A previous study demonstrated that approximately 80% of lung cancers are non-small cell lung cancer (NSCLC), 50–55% of which are lung adenocarcinoma (LUAD) (Relli et al., 2019). Despite improvements in primary prevention gaining more attention in recent years, LUAD is still difficult to diagnose at an early stage due to the delayed occurrence of symptoms. Recently, an accumulating collection of research indicates that abnormal cancer metabolism has a critical role in cancer metastasis, immune escape, and drug resistance. Proteins, lipids, and nucleic acids are the most common macromolecular classes affected in cancer metabolism, as biosynthesis of all three classes is activated in tumorigenesis (Bader et al., 2020; Bose et al., 2020; Dong et al., 2020; Navas and Carnero, 2021; Yang et al., 2021). Thus, the regulatory mechanisms that regulate cancer metabolism have attracted great attention as a prognostic marker and a potential therapeutic target.

Tumor microenvironment (TME) is closely related to tumor progression. At present, more and more studies show that the changes of tumor microenvironment may be related to abnormal tumor metabolism. Immunotherapy targeting immune checkpoint proteins, such as CTLA-4 and PD-1/L1, has benefited a growing number of patients (Kordbacheh et al., 2018; Zhao et al., 2019; Liu and Zheng, 2020). The infiltration of immune cells into TMEs is a key factor in determining the efficiency of immunotherapy. Furthermore, the production of immune-suppressive metabolites can dampen the antitumor activity of immune cells and promote tumor immunity escape by affecting the expression of cell surface markers (Marin-Acevedo et al., 2021; Memmott et al., 2021; Qiao et al., 2021). Immune checkpoint blockades, such as PD1 and B7-H3, can restore glucose in the TME and permit T cell glycolysis and cytokine production. Immune responses can also be fostered by targeting tumor-intrinsic metabolism (Noël et al., 2018). Although targeting metabolism, such as glutamine metabolism and hexosamine biosynthesis, cannot suppress or activate the immune system completely as it still selectively regulates immune responses (Byun et al., 2020; Lam et al., 2021). Immune checkpoint inhibitors combined with targeting metabolism might be a novel strategy to overcome the immune resistance in immunotherapy. Therefore, comprehensively understanding the effect of tumor metabolism on immune cell infiltration within TME will be helpful for the development of new therapy.

In previous studies, all prediction models were constructed based on RNA-seq data, which focused on only single metabolic genes or immune-related genes, failing to consider the influence of multiple factors. In this study, we used Multivariate Cox Proportional Hazards Regression Analysis to construct an optimized metabolic-immune protein risk score (MIPRS) model based on the protein expression of metabolic and immune proteins to classify LUAD into two subtypes with discrete survival rates. Compared to previous biomarkers, MIPRS is a technically simple and reliable tool to predict the prognosis of LUAD patients.

## Materials and Methods

### Data Acquisition

We downloaded the gene expression matrix and clinical information of 517 LUAD samples and 59 non-tumor samples from The Cancer Genome Atlas (TCGA) database (https://portal.gdc.cancer.gov/repository) as the training set. We then downloaded 266 LUAD samples from the microarray dataset GSE31210 (http://www.ncbi.nlm.nih.gov/geo/) as the first validation set. GSE135222, produced by Illumina HiSeq 2500, was also downloaded from GEO for pharmacodynamic evaluation. In addition, we used 50 LUAD patients from the Xiangya Hospital Central South University as the second validation set, the clinical information of which is shown in Supplementary Table S1. The data of metabolizing enzyme genes was downloaded from the KEGG PATHWAY database (https://www.genome.jp/kegg/pathway.html). The abundance of 22 immune cells infiltration were calculated by CIBERSORT.

### Multivariate Cox Proportional Hazards Regression Analysis

Multivariate Cox proportional hazards regression analysis utilized by the R package “survival” and “survminer” was performed to screen suitable biomarkers. Subsequently, TYMS, NME4, LDHA and SMOX were finally selected as four key metabolic enzyme genes. According to the regression coefficients of four genes, we established a model in the training set to calculate the risk score of LUAD patients. The formula of risk score (metabolic genes risk score (MGRS)) is as follows:

MGRS=0.28 ×TYMS+ 0.25×NME4+0.41×LDHA+0.20×SMOX

### Immunohistochemical Assay

Paraffin-embedded lung tissue sections (5-µm) were prepared. After conventional dewaxed to water, the sections were boiled in a pressure cooker with EDTA buffer (pH 8.0) for 2.5 min at 125°C. We then incubated the sections in 5% BSA for 1 h at 37°C. Sections were incubated with primary rabbit anti-LDHA antibody (19987-1-AP; dilution, 1:100; proteintech), anti-TYMS antibody (15047-1-AP; dilution, 1:100; proteintech), anti-SMOX antibody (15047-1-AP; dilution, 1:100; proteintech), anti-NME4 antibody (a8350; dilution, 1:100; ABclonal), anti-CD19 (MAB-0705, MaiXin Biotechnologies, Fuzhou, China) andCD68 (Kit-0026, MaiXin Biotechnologies, Fuzhou, China) at 4°C overnight. The expression of the four enzymes was shown in brown. In order to achieve the purpose of semi quantitative staining, the number of positive cells in each section and their staining intensity were converted into corresponding values. The Mantra™ quantitative pathology workstation, with inForm® image analysis software, were used to analyze the results of immunohistochemistry. The scores of 0, 1, 2 and 3 represent no staining, weak staining, moderate staining and strong staining of target cells, respectively. The sum of percentage of staining scores was calculated to get histochemical score (H-SCORE). Each slice were randomly observed three horizons to calculate the score and took the average as the final score. The median value of H-score was used to distinguish high expression and low expression samples.

### Statistical Analysis

All the analysis process was performed by the Strawberry Perl (version 5.32.1.1) software and R (version 4.0.4) software. We have log2 transformed all the data before all of the analysis. The Wilcoxon test and chi-square test were performed for comparisons between two groups and three or more groups respectively. We used both the Kaplan-Meier analysis and Univariate Cox analysis to screen the metabolic enzyme genes negatively correlated with the overall survival (OS). Kyoto Encyclopedia of Genes and Genomes (KEGG) pathway analysis and Gene Ontology (GO) analysis were performed to find differentiation of metabolic pathways in low and high risk groups.

## RESULTS

### Identification of Differentially-Expressed Metabolic Genes in Lung Adenocarcinoma

**FIGURE 1:**
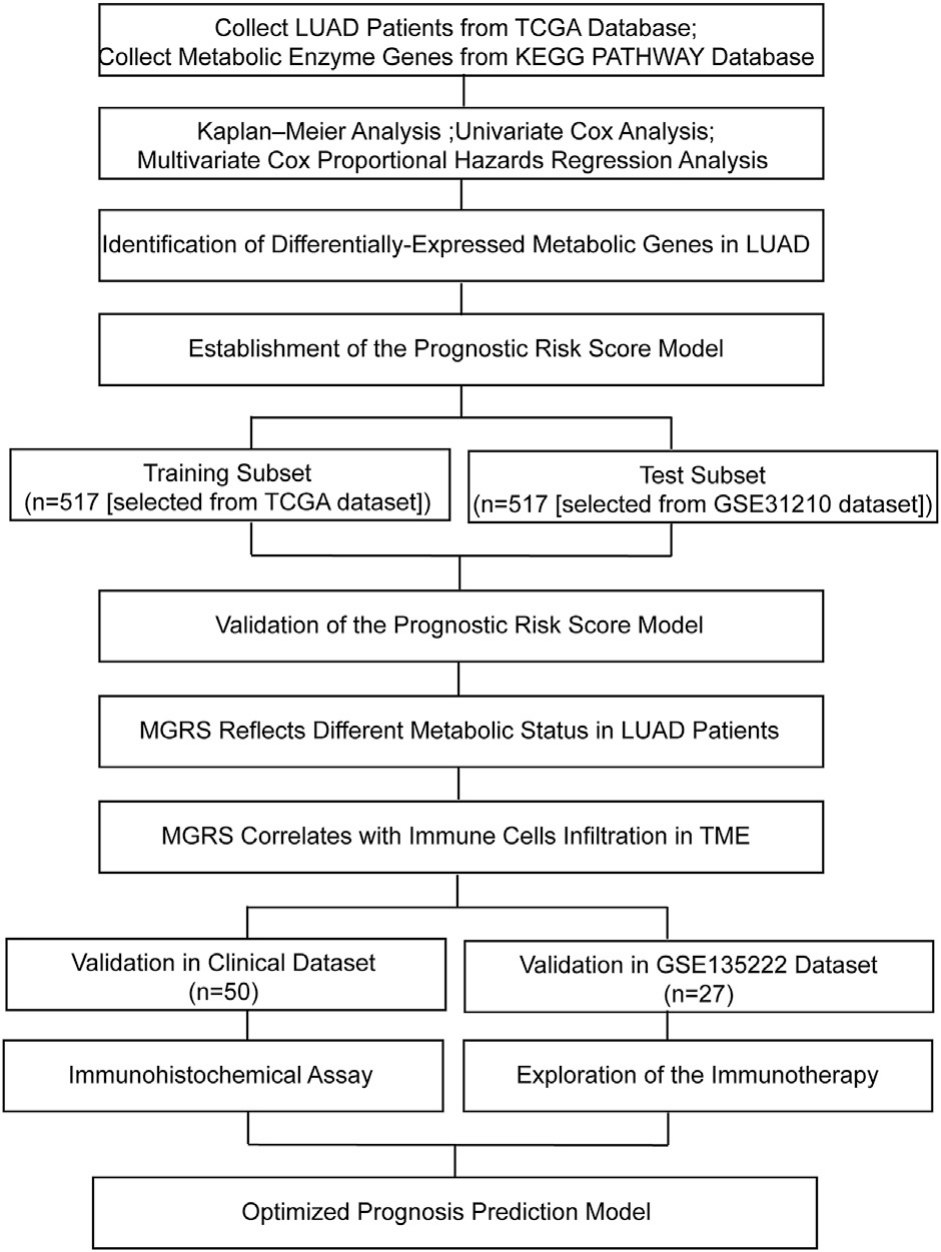
Flow chart of this study. LUAD, lung adenocarcinoma; MGRS, metabolic genes risk score; TME, tumor microenvironment.

**FIGURE 2:**
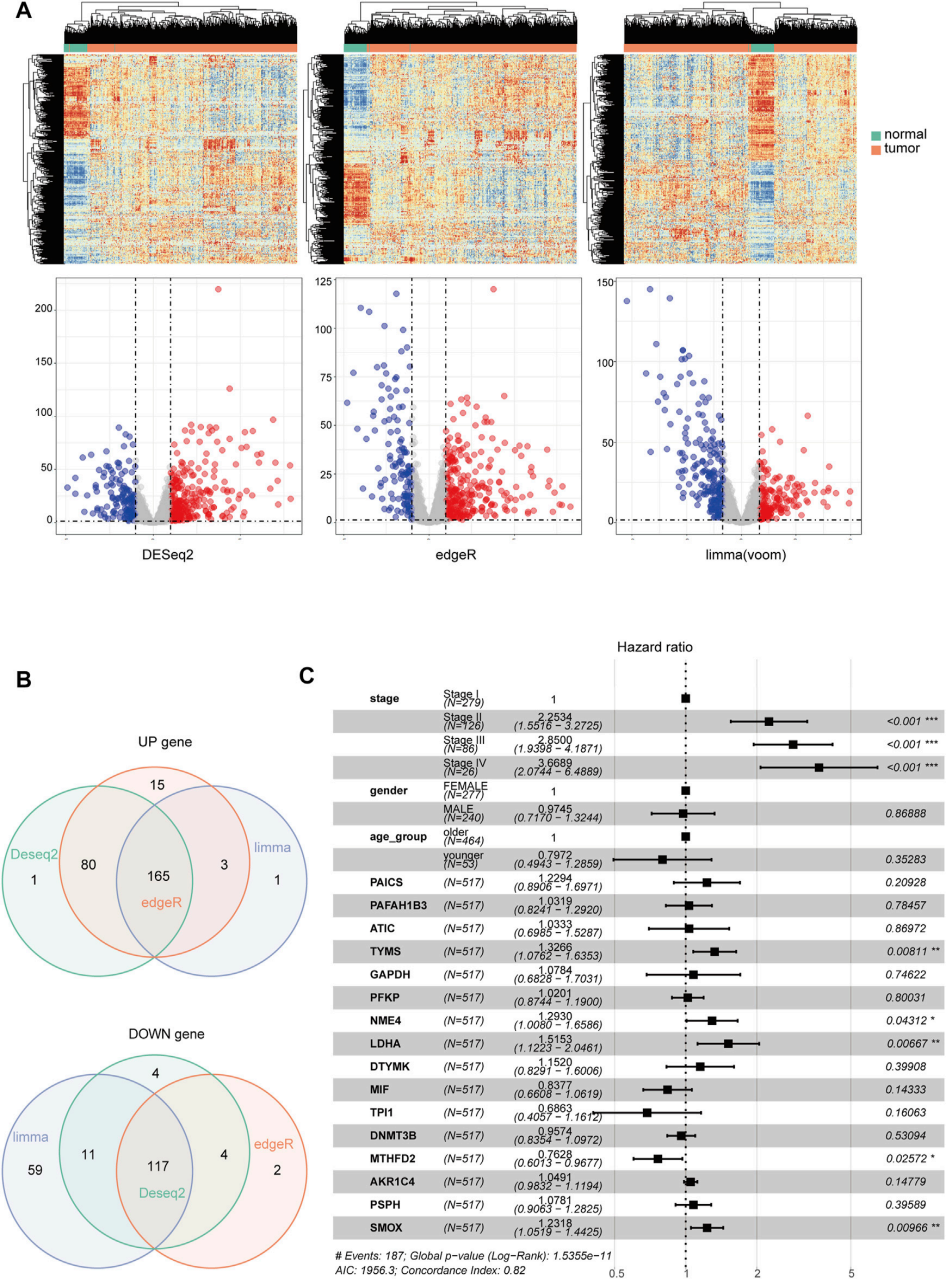
The identification of differentially-expressed metabolic genes. Heatmap and Volcano Plot **(A)**, Venn Diagram **(B)** compared the results of 3 R packages ‘DESeq2,’ ‘edgeR,’ and ‘limma’. respectively. **(C)** A forest map showed 16 metabolic enzyme genes identified by Multivariate Cox Proportional Hazards Regression analysis. Significance codes: <0.001 = ∗∗∗, <0.01 = ∗∗, and <0.05 = ∗.

The overview of the process used in our study is shown in Figure 1. First, we downloaded the expression profile datasets from the TCGA database; 517 LUAD samples and 59 non-tumor samples were included. A total of 1399 metabolic enzyme genes, which were based on the list from the KEGG PATHWAY database, were selected to analyze the distinct expression between LUAD and the adjacent normal samples. 282 metabolizing enzyme genes, with a threshold P-value < 0.05 and |logFC| > 1, were distinguished by using 3 R packages “DESeq2,” “edgeR,” and “limma,” among which 165 genes were determined to be upregulated and 117 downregulated. The R package “pheatmap” and “ggplot2” were performed to draw the heatmap, volcano plots and Venn Diagram, which were shown in Figures 2A,B respectively.

### Establishment and Validation of the Prognostic Risk Score Model

Kaplan-Meier analysis and Univariate Cox analysis utilized by the R package “survival” and “survminer” was performed to screen 165 upregulated metabolic genes. We used Log Rank test to evaluate the results of survival analysis. There were significant differences in the genes with *p* < 0.05 by Log Rank test. The number of significant genes was 31 by Kaplan-Meier analysis and 24 by univariate Cox analysis. We finally selected the union of the two analysis results, and a total of 34 valid genes were identified as risk factors of overall survival rate in LUAD patients. After checking the immunohistochemical results of the HPA database (https://www.proteinatlas.org/), only 16 genes, which showed significant overexpression in cancer tissues, were extracted for further Multivariate Cox Proportional Hazards Regression Analysis (Figure 2C). Four metabolic genes with statistically significant differences, namely TYMS, NME4, LDHA, and SMOX, were finally identified. We extracted these four genes separately for another Multivariate Cox Proportional Hazards Regression Analysis and get their regression coefficients from the forest map (Supplementary Figure S1A ). According to the regression coefficients of these four genes, we established a model in the TCGA training set to calculate the risk score of LUAD patients. This risk score, referred to hereafter as the metabolic gene risk score (MGRS), was calculated as follows:

MGRS=0.28×TYMS+0.25×NME4+0.41×LDHA+0.20×SMOX

**FIGURE 3:**
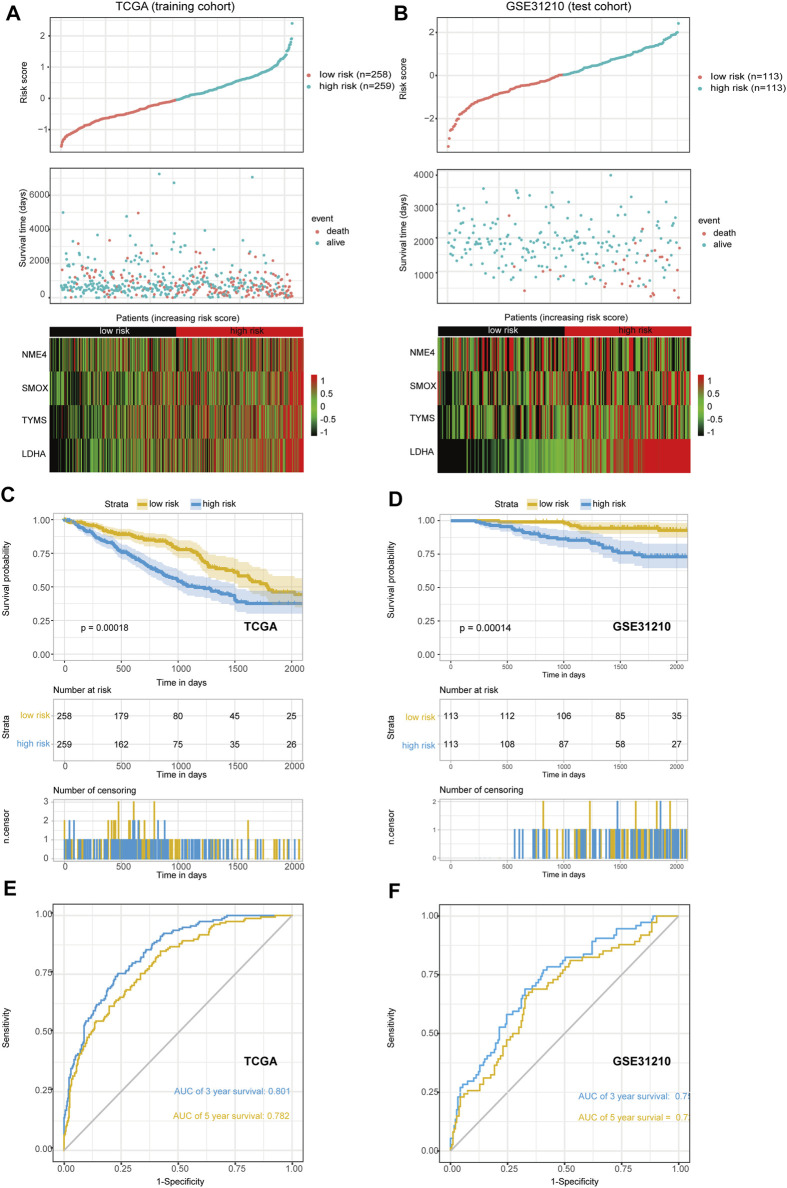
The scatter plot of risk score and heat map of four prognostic genes. **(A,B)** The training set **(A)** and the first validation set **(B)** are shown. **(C,D)** Kaplan-Meier curves of overall survival (OS) for patients with LUAD based on the risk score. The training set **(C)** and the first validation set **(D)** are shown **(E,F)** The 3-, and 5-years ROC of the risk model. The training set **(E)** and the first validation set **(F)** are shown.

The median risk scores were considered as the cutoff value to classify patients into low-risk and high-risk groups. To test the model predictions, we used scatter plots and heat maps to determine a rough estimate of whether the score of this formula can distinguish the prognosis of patients (Figures 3A,B) in both the training and test cohorts. The results suggested that all of the prognostic genes are risky genes and people with high scores from our model might be associated with poor outcomes. We then performed a Kaplan-Meier analysis to validate this cut-off point and show the survival difference between the high-risk and low-risk groups (Figures 3C,D). We also performed time-dependent receiver operating characteristic (ROC) curve analysis using the R package “survivalROC” both in the training and first validation set. The areas under the ROC curve at 3 and 5 years were 0.801 and 0.782in the training set and 0.750 and 0.721 in the first validation set, respectively (Figures 3E,F). These results showed that the MGRS had a powerful prognostic performance of survival value for LUAD.

### Metabolic Gene Risk Score Reflects Different Metabolic Status in Lung Adenocarcinoma Patients

**FIGURE 4:**
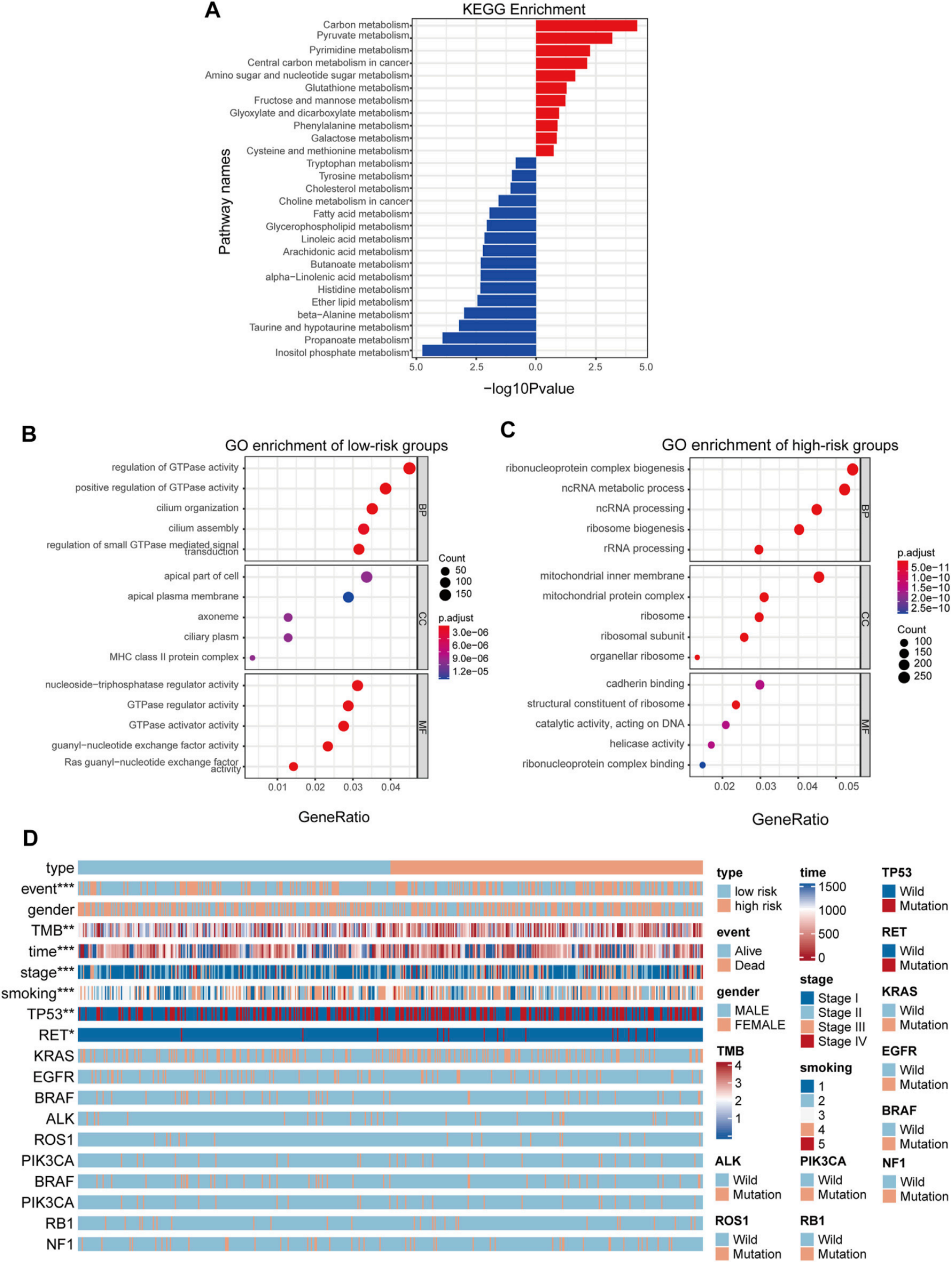
MGRS can identify the survival status of different patients. **(A)** KEGG pathway enrichment analyses for differentially-expressed metabolic genes **(B,C)** GO pathway enrichment analyses for differentially-expressed genes. Pathways activated in low-risk groups **(B)** Pathways activated in high-risk groups **(C)** are shown. All enriched pathways were significant. **(D)** A heatmap showed that clinical stage, survival status, smoking status, Tumor Mutational Burden (TMB), and gene mutations in TP53 and RET were significantly associated with risk.

As the prognostic value of the MGRS indicated that this 4-genes-based signature might reflect the distinct metabolic status of LUAD progression, we next performed Kyoto Encyclopedia of Genes and Genomes (KEGG) pathway analysis via the R package “clusterProfiler” to identify differences in metabolic pathway enrichment in low- and high-risk groups. We found that carbon metabolism and nucleotide metabolism were significantly activated in high-risk groups, while some pathways such as inositol phosphate metabolism and propanoate metabolism were inhibited (Figure 4A). In order to further study the effects of metabolic differences on the molecular pathways of tumor cells, we used Gene Ontology (GO) to analyze the differentially expressed genes between the two groups. The results showed that GTPase pathway was activated in low-risk group (Figure 4B), while ribonucleoprotein complex biogenesis pathways were activated in high-risk groups (Figure 4C). To assess the clinical application value of the constructed model, a Wilcoxon test and chi-square test of clinicopathological characteristics were performed and visualized by a heat map, labeled as *p* < 0.001 = ∗∗∗, *p* < 0.01 = ∗∗ ∗, and *p* < 0.05 = ∗. The results showed that clinical stage, survival status, smoking status, Tumor Mutational Burden (TMB), and gene mutation of TP53 and RET were significantly correlated with the level of LUAD risk (Figure 4D). Patient’s original clinical information and individual p-value for each clinical character are shown in Supplementary Tables S2, S3.

### Metabolic Gene Risk Score Correlates With Immune Cells Infiltration in Tumor Microenvironment

**FIGURE 5:**
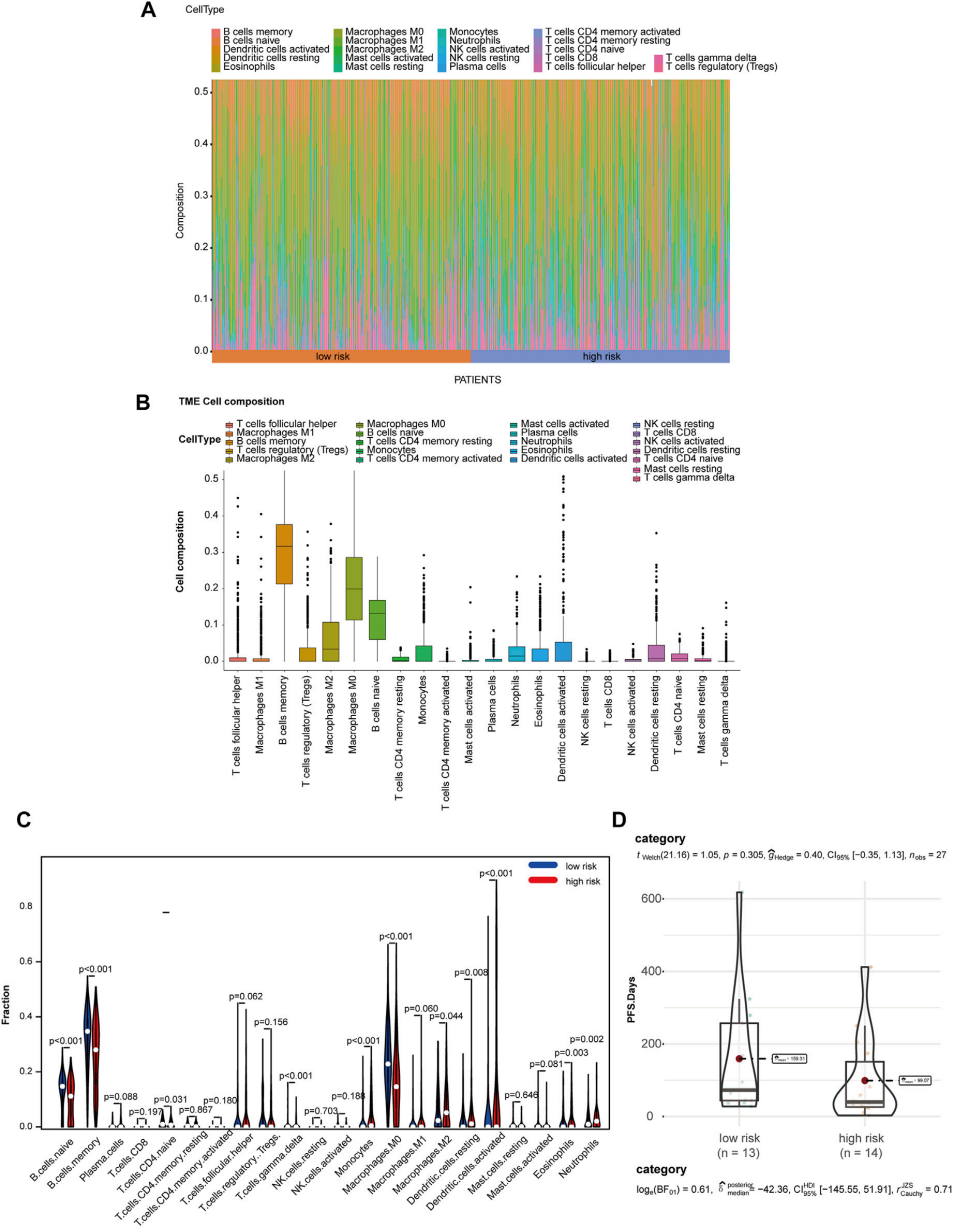
MGRS was associated with TME immune cell infiltration. **(A)** Differential distribution of immune cells in each patient. **(B)** Differential distribution of immune cells in all patients. **(C)** Differential distribution of immune cells in low- and high-risk groups. **(D)** Box/violin plots showing the differentiation of the IC50 of anti-PD-1/L1 antibodyin low- and high-risk groups.

Due to the crucial role of tumor metabolism in remodeling the TME, we analyzed the abundance of infiltration by 22 types of immune cells by CIBERSORT to evaluate the correlation of MRGS and immune cell infiltration in TME. Differential distribution of immune cells in all patients is shown in Figures 5A,B. We found that the high-risk group exhibited significantly decreased B cell and increased M2 macrophage infiltration (Figure 5C). To analyze the relationship between the model and immunotherapy, we calculated the IC50 of the immune checkpoint inhibitor PD1 obtained from the microarray dataset GSE135222. A Wilcoxon test was performed to analyze differentiation of the IC50 of anti-PD-1/L1 antibody in the low- and high-risk groups (Figure 5D). We found that patients with high-risk scores exhibited low therapeutic efficacy compared to low-risk patients, although these results were not statistically significant due to the limited sample size.

### Optimized Prognosis Prediction Model Based on Immunohistochemistry

**FIGURE 6:**
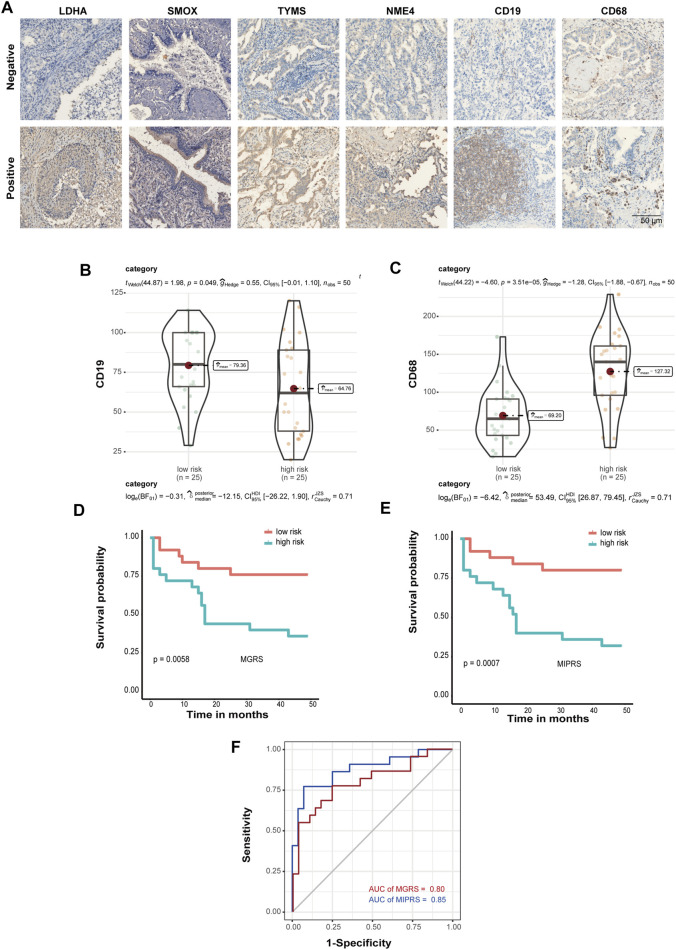
Prediction model optimization based on immunohistochemistry. **(A)** The representative IHC positive and negative images of four prognostic proteins, as well as markers of B cells (CD19) and macrophages (CD68). Box/violin plot showing that the expression of CD19 **(B)** and CD68 **(C)** was significantly correlated with the risk score. **(D,E)** Kaplan-Meier curves of the prediction model. The original model **(D)** and the optimized model **(E)** are shown. **(F)** ROC curves of the original model and the optimized model.

To further validate the prognostic power of these four metabolic enzymes in LUAD and to create a technically simple tool applied in clinical diagnosis, we performed immunohistochemical analysis to verify the expression levels of the proteins encoded by these four genes, as well as that of the markers of B cells (CD19) and macrophages (CD68) in a second validation set. The representative IHC positive and negative images are shown in Figure 6A. The resulting H-score is shown in Supplementary Table S4. According to the MGRS model formula and H-score of four metabolic genes, the samples were also divided into low-risk (*n* = 25) and high-risk groups (*n* = 25) that exhibited a significantly different survival rate. Furthermore, we found that the level of CD19 and CD68 were significantly correlated with the risk score (Figures 6B,C). Therefore, we included CD19 and CD68 in Multivariate Cox Proportional Hazards Regression Analysis as well (Supplementary Figure S1B). We constructed an optimized model (the metabolic-immune protein risk score (MPIRS)) by combining the expression data of the four metabolic genes and immune cells as follows:

MPIRS=0.12 ×TYMS+ 0.09×NME4+0.23×LDHA+0.12×SMOX+0.12×CD68−0.04×CD19

Kaplan Meier and ROC curve analyses were performed using the original model and optimized model, respectively (Figures 6D–F). The results show that the optimized model can evaluate the survival prognosis of patients more accurately, which indicated that these models are useful for clinical diagnosis.

## Discussion

In this study, a metabolic enzyme signature model referred to as MGRS was constructed to predict the overall survival of LUAD patients and distinguish patients into low-risk and high-risk groups. Using multivariate Cox proportional hazards regression analysis, four metabolic enzymes (*TYMS, NME4, LDHA, and SMOX*) were identified as ideal prognostic markers. Kaplan Meier analysis and ROC curve analysis were performed both in the training set and the first validation set. We revealed the ribonucleoprotein complex biogenesis pathway was activated in high-risk patients while GTPases were up-regulated in low-risk groups, which may mediate immune escape and confer different outcomes in patients. To evaluate the model in the immune-cell infiltration, we performed the Wilcoxon test to analyze the infiltration of 22 immune cells via CIBERSORT. These results show that high-risk patients suffered a lower survival rate with significantly decreased B cells and increased macrophages M2 infiltration of the TME. In the subsequent analysis, we calculated the risk scores in a cohort with anti-PD-L1immunotherapy. The predictive accuracy of the signature was also confirmed by immunohistochemical assays. We finally constructed an optimized MIPRS model based on the expression of metabolic and immune proteins, which is useful for accurately predicting patient prognosis and should serve as a useful tool in clinical diagnosis.

In recent years, the role of abnormal metabolism in tumor progression has attracted more and more attention. Studies have demonstrated that compared to traditional biomarkers, metabolic enzymes are superior as predictive biomarkers for various cancers (Dong et al., 2015; Xu et al., 2021). Metabolic alteration has wide-ranging effects, such as angiogenesis, metastasis, and immune escape, which has provided opportunities to solve immunotherapy resistance and poor clinical outcomes (Broadfield et al., 2021; Jones et al., 2021; Zanotelli et al., 2021). To date, the expression patterns of metabolic enzymes have been widely studied in different cancer types, such as liver, gastric, colorectal, breast, and prostate cancer (Lavorgna et al., 2018; Zinger et al., 2019; Rivello et al., 2020; Sun et al., 2020; Xu et al., 2020; Jiang et al., 2021). With the development of next-generation sequencing, we can use multiple appropriate statistical methods to analyze TME cell infiltration mediated by metabolic enzymes. However, most of these metabolism-related gene signatures included one or two key molecules and TME infiltration cells, ignoring the fact that metabolic regulation is a complex and interdependent network. In addition, these signatures are based on mRNA levels, which made application to clinical diagnoses difficult. To identify the comprehensive role of metabolic enzymes in heterogeneous and complex processes such as TME cell infiltration, the construction of an integrated metabolic enzyme signature based on immunohistochemical analysis might address the aforementioned limitations.

Metabolic enzymes in our study have been investigated in various cancers. A previous study demonstrated that Lactate dehydrogenase A (LDHA) functions as a sensor for overloaded ROS to enhance antioxidant capacity and sustain cell proliferation by producing α-HB in the nucleus (Liu et al., 2018). Another study showed that the loss of DNA methylation of LDHA was associated with certain malignant clinicopathological features such as a high glycolytic phenotype in gliomas (Ruiz-Rodado et al., 2020). LDHA was also associated with a poor prognosis in pancreatic cancer by regulating the expression of L-2 hydroxyglutarate, an epigenetic modifier, which can inhibit T cell proliferation and migration and thereby contribute to immune escape (Gupta et al., 2021). Thymidylate synthase (TYMS) is a key dNTP synthesizing enzyme and regulates nucleotide metabolism through a YBX1-RRM2-TYMS-TK1 axis in the liver, breast, and lung cancer (Gandhi et al., 2020). The expression of TYMS can be suppressed by inhibiting the synthesis of hydrogen sulfide (H2S) and might be used as a potential therapeutic target to reverse the acquired resistance to 5-FU in colon cancer cells (Ahn et al., 2015). Nucleoside Diphosphate Kinase 4 (NME4) is an enzyme regulating nucleotide metabolism and ATP/ITP metabolism as well (Lu et al., 2014). NME4 is involved in apoptosis and inflammatory reactions through an NME4/NDPK-D-based CL-transfer pathway (Schlattner et al., 2018). Since there are no reports of NME4 associated with the development of any cancer, it might be a novel prognostic signature in LUAD. Spermine oxidase (SMOX) is potentially associated with oxidative DNA damage in gastric cancer by catabolizing polyamine spermine and producing H_2_O_2_ (Chaturvedi et al., 2014; Chaturvedi et al., 2015; Murray-Stewart et al., 2016; Sierra et al., 2020). Although no studies have reported the role of SMOX in LUAD, it has a significant correlation with chronic inflammation, such as in ulcerative colitis, prostatic intraepithelial neoplasia (PIN), and *Helicobacter* pylori-associated gastritis (Goodwin et al., 2008; Hong et al., 2010; Chaturvedi et al., 2014). Accordingly, all of these enzymes are involved in tumor-related biological processes.

The results of KEGG and GO pathway enrichment analyses in our study show that the formula determined by these four metabolic genes can effectively distinguish different types of patients, high-risk and low risk patients have significant differences in metabolic pathway. GO enrichment results showed that GTPase pathway was activated in low risk group, while ribonucleoprotein complex biosynthesis pathway was activated in high risk group. GTPase-activating protein (GAP), also known as RGS protein or RGS protein, plays an important role in controlling the activity of G protein, involving in cell proliferation, differentiation, survival and movement (Moon and Zheng, 2003; Gray et al., 2020). GTPase activating protein can bind to the activated G protein and stimulate its GTPase activity, thus terminating the signal. Mutations in GTPase are closely related to carcinogenesis (Thaker et al., 2019; Zhou et al., 2020). Similar to the results of KEGG analysis, the increase of ribonucleoprotein complex biogenesis was the main factor in the high risk group. In the KEGG analysis, we also found that carbon metabolism, ribonucleoprotein complex biogenesis pathways and sugar metabolism are activated in the LUAD high-risk group. Carbon metabolism is crucial for oxidative phosphorylation in cells (Ciccarone et al., 2017). Current studies have shown that cancer cells are more active in anaerobic glycolysis while the tricarboxylic acid (TCA) cycle plays an important role in regulating energy production in normal cells (Montal et al., 2015; Anderson et al., 2018; Cai et al., 2020). One of our candidate enzymes, LDHA, is key for anaerobic respiration and catalyzes the inter-conversion of pyruvate and L-lactate using NADH (Ooi and Gomperts, 2015). Our study also found that the frequency of TP53 gene mutation in high-risk score patients is significantly higher than that in low-risk score patients. TP53 can inhibit tumor growth and development by not only downregulating glucose transporters (GLUT1 and GLUT4) but suppressing the activity of glycolytic enzymes, which was verified in the constructed model (Duffy et al., 2020; Huang, 2021; Liu et al., 2021). Except for carbohydrate metabolism, nucleotide metabolism is involved in the progression of various tumors as well. The carbon metabolism mentioned above also plays an important role in nucleotide synthesis and biomethylation(Newman et al., 2021). Folate mediated carbon metabolism transfers a part of carbon (methyl) to many biological reactions and plays a variety of important roles in normal and abnormal cell division. For example, the synthesis of purine bases, adenine and guanine requires the participation of 10-formyltetrahydrofolate(Hong et al., 2020). Another enzyme of our model, TYMS, served as a key enzyme in pyrimidine metabolism, provides the sole *de novo* pathway for the production of deoxythymidine monophosphate (dTMP), which is a component of DNA (Rosmarin et al., 2015). Mutant TP53 also controls the abundance of deoxyribonucleotides by regulating the abundance of ribonucleotide reductase (RNR) (Xue et al., 2003; Zhou et al., 2003; Link et al., 2008). Although SMOX is a key enzyme of polyamine metabolism, it may still indirectly regulate pyrimidine metabolism by consuming purine during polyamine synthesis (Fan J. et al., 2019). Meanwhile, photoaffinity polyamines can change the helical twist of DNA in nucleosomes by regulating their affinity for DNA, which may, in turn, trigger tumor development (Amarantos and Kalpaxis, 2000). At present, there are few studies on the abnormal metabolism of B cells in tumor, but B cells may also affect the metabolism of tumor immune microenvironment by cooperating with T cells and NK cells.

To evaluate our model of immune-cell infiltration, we performed the Wilcoxon test to analyze the infiltration of 22 types of immune cells using CIBERSORT. These results showed that the high-risk group suffered a poor survival rate and had significantly decreased B cell and increased M2 macrophage infiltration. M2 macrophages are associated with malignant transformation and metastasis in many cancers (Fan CS. et al., 2019), indicating the poor prognosis of the high-risk group. Compared with other inflammatory macrophages, tumor M2 macrophages prefer unique arginine metabolism. Arginine metabolism is the key pathway of innate and adaptive immune response (Luo et al., 2018; Das et al., 2019). After the death of immune cells, they are released from phagocytic lysosomes and consume arginine in the microenvironment, thus inhibiting the proliferation of T cells and natural killer (NK) cells and the secretion of cytokines(Munder, 2009). Arginase 1 (ARG1) is a key enzyme in the urea cycle, which converts L-arginine into urea and L-ornithine. Tumor M2 macrophages produce more polyamines and consume arginine by increasing ARG1 expression(De Santo et al., 2018; Baier et al., 2020). The spermine oxidase (SMOX) in our model is also one of the key enzymes in arginine metabolism, which can decompose polyamine and spermine, and may also participate in the metabolism of M2 macrophages. In most cases, B cells serve as anticancer cells by inducing antigen-specific CD4^+^ and CD8^+^ T-cell responses as antigen-presenting cells, with the exception of B-cell lymphoma (Menon et al., 2021; Michaud et al., 2021). In the subsequent analysis, we calculated the risk scores in a cohort treated with anti-PDL1 immunotherapy. PD-L1 (Programmed cell death 1 ligand 1), a ligand of PD-1 (Programmed cell death-1) expressed mainly on tumor cells in the TME, can suppress the function of cytotoxic T cells (Doroshow et al., 2021). In our study, patients with high-risk scores exhibited low therapeutic efficacy compared to low-risk patients, although these results were not statistically significant due to the limited sample size. Although previous reports have shown that anti-PDL1 immunotherapy mostly regulates T cells, tumor-infiltrating B cells (TIL-B) are also involved in the regulation of this process. Compared to spleen B cells, the expression of PD-L1 in TIL-B cells was significantly increased, which might be due to the reduction of calcium signaling (Ou et al., 2014) (Schwartz et al., 2016). TIL-B cells may inhibit the proliferation and immune response of T cells by increasing the expression of PDL1 (Zhang et al., 2016; Nus et al., 2020). Studies have shown that PDL1 antibodies have a promising therapeutic effect on EMT-6 mammary carcinoma with significantly infiltrated B cells (Schwartz et al., 2016). Therefore, the sensitivity of low-risk patients to PDL1 therapy may be attributed to the increased numbers of TIL-B cells.

Finally, we optimized our predictive model (MIPRS) based on the protein level of metabolic enzymes and immune cell markers, by which we can stratify patients into two types according to their calculated risk score. Compared to previous biomarkers, MIPRS is technically simple and might be more accurate in predicting LUAD prognoses. Accordingly, the predictive power of this signature should be assessed in more cases in the future.

## Data Availability

The original contributions presented in the study are included in the article/Supplementary Material, further inquiries can be directed to the corresponding author.
