# Supplementary figures and images for: A Novel Integrated Metabolism-Immunity Gene Expression Model Predicts the Prognosis of Lung Adenocarcinoma Patients
Source: Front Pharmacol. 2021 Jul 30;12:728368. doi: 10.3389/fphar.2021.728368 (PMC8361602; doi:10.3389/fphar.2021.728368)

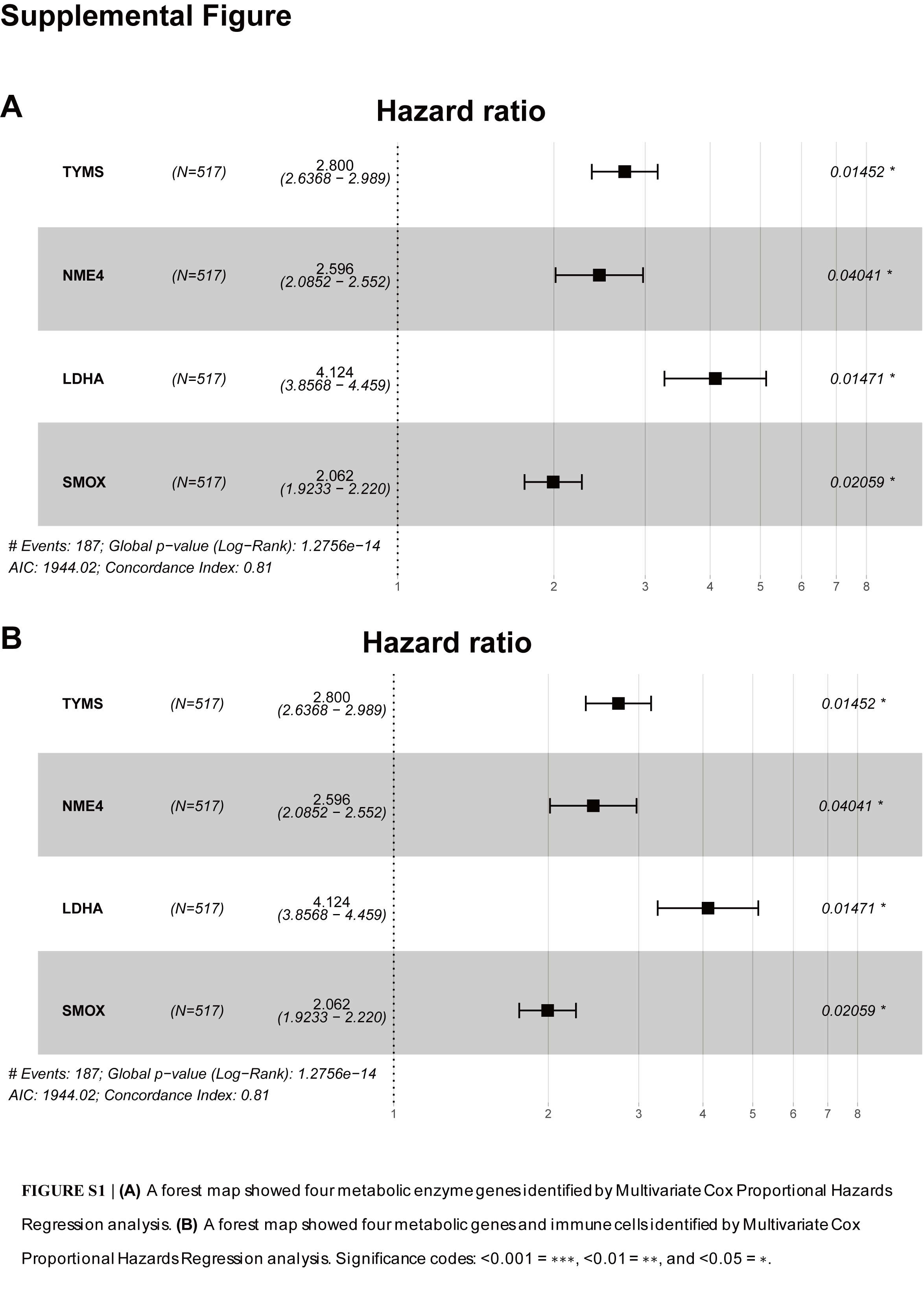

Supplement: Supplementary file 3 [file Image1.TIF]
